# Association between methylenetetrahydrofolate reductase gene rs1801131 A/C polymorphism and urinary tumors’ susceptibility

**DOI:** 10.1186/s41065-020-00129-x

**Published:** 2020-04-27

**Authors:** Shuaili Xu, Li Zuo

**Affiliations:** 1 Department of Paediatrics, Changzhou No. 2 People’s Hospital Affiliated to Nanjing Medical University, Changzhou, 213003 Jiangsu Province China; 2 Department of Urology, Changzhou No. 2 People’s Hospital Affiliated to Nanjing Medical University, Changzhou, 213003 Jiangsu Province China

**Keywords:** MTHFR, rs1801131, Prostate, Bladder, Renal, Polymorphism, Risk

## Abstract

**Background:**

The methylenetetrahydrofolate reductase (MTHFR) rs1801131 A/C variant results in a decrease in MTHFR enzymatic activity, which may play an important role in folate metabolism and is also an important source of DNA methylation and DNA synthesis. Several case-control studies have been conducted to assess the association of MTHFR rs1801131 polymorphism with the risk of urinary cancers, yet with conflicting conclusions. To derive a more precise estimation of above relationship, the association between the MTHFR rs1801131 A/C polymorphism and the risk of urinary cancer was performed.

**Methods:**

A total of 28 case-control studies was identified. The odds ratios (OR) with 95% confidence intervals (CI) was calculated to assess.

**Results:**

On one hand, we found that the MTHFR rs1801131 A/C polymorphism was associated with increased whole urinary cancers’ risk (for example CA vs. AA: OR = 1.12. 95%CI = 1.01–1.24). On the other hand, we found that the MTHFR rs1801131 A/C polymorphism might increase bladder cancer risk both in Asian (C-allele vs. A-allele: OR = 1.35. 95%CI = 1.15–1.60) and African populations (CA vs. AA: OR = 1.63. 95%CI = 1.17–2.25).

**Conclusions:**

Our current analysis suggested that MTHFR rs1801131 A/C is associated with urinary cancers, especially bladder cancer.

## Background

Previous epidemiological studies have shown an association between low folate intake and an increased urinary cancer risk [1, 2], meanwhile, folate deficiency may increase cancer risk through impaired DNA repair synthesis and disruption of DNA methylation, which may participate in cancer development [3, 4]. Methylenetetrahydrofolate reductase (MTHFR) plays a crucial role in the metabolism of folates and converts irreversibly 5,10-methylenetetrahydrofolate (5,10-MTHF) to 5-MTHF, which is the predominant circulatory form of folate and donates a metyl group for the re-methylation of homocysteine to methionine. Then, the methionine is metabolized to yield S-adenosylmethionine (SAM), which is the main methyl donor for vital methylation reactions and is required for DNA repair [5, 6]. In summary, this gene could influence cancer development.

A common single nucleotide polymorphism (SNP), A1298C/rs1801131 A/C, is located in the coding carboxy-terminal regulatory region domain [7] and lymphocytes from individuals containing 1298CC genotype have been found to have approximately 60% of wild-type in vitro MTHFR activity [8], which acts as a risk factor in cancer development.

Previous studies have investigated that MTHFR rs1801131 A/C was involved in the development of urinary cancers. However, the results of these studies remain conflicting. With the aim to measure the correlation, we performed this comprehensive meta-analysis by adopting all eligible studies [9–34].

## Methods

### The search strategy

We searched the Pubmed database (updated on Sep 10, 2018), using combinations of the keywords: ‘polymorphism,’ or ‘variant’ or ‘mutation’ and ‘bladder cancer’ or ‘prostate cancer’ or ‘renal’ and ‘MTHFR’ or ‘methylenetetrahydrofolate reductase’. All the included studies met the following criteria (1) the association between MTHFR rs1801131 A/C and urinary cancer risk was evaluated; (2) case-control studies were designed; (3) available genotype frequency was collected. The major exclusion criteria were (1) duplications; (2) insufficient reporting data; (3) abstract, commentary, review, editorial article and conference article.

### Data extraction

Two authors carefully extracted data from all eligible publications, independently. The following data were collected from each study: first author’s last name, year of publication, race of origin, cancer type, sample size (cases/controls), study design (hospital-based, HB, or population-based, PB), source of control for prostate cancer subgroup, Hardy-Weinberg equilibrium (HWE) of controls and genotype method.

### Quality score assessment

The Newcastle-Ottawa Score (NOS) were selected to assess the quality of each study and to assess the various aspects of the methodology used by the observational research, which are relevant to the quality of the study, including the selection of cases, the comparability of groups and the determination of exposure. The total score is from 0 to 9 star. Studies with scores more than 7 are to be as high quality [35].

### Statistical analysis

Odd ratio (OR) with 95% confidence interval (CI) was used to measure the strength of the association between rs1801131 A/C and urinary cancers. Four different genetic models were applied to evaluate above association: allelic contrast (C-allele vs. A-allele), heterozygote comparison (CA vs. AA), dominant genetic model (CC + CA vs. AA), and recessive genetic model (CC vs. CA + AA). The ethnic descents were categorized as Caucasian, Asian, African, or Mixed. The control group based on sources was divided as follows: HB, PB, benign prostatic hyperplasia (BPH), and healthy man.

The statistical significance of the summary OR was determined with the *Z*-test. The heterogeneity was evaluated by both Cochrane *Q*-test [36, 37] and *I*^2^ metric [38, 39] ranging from 0 to 100%. When *P* for the heterogeneity test (*P*_*h*_) < 0.10 and *I*^2^ > 50% [40], the pooled OR of each study was calculated by using the random-effects model; otherwise, the fixed-effects model was used [41, 42].

Subgroup analysis was performed according to the ethnicity and the source of cases to explore potential heterogeneity. The meta-regression analysis is a technique used to assess heterogeneity between the studies [43]. This statistical approach determines whether there is a significant association between the study period and number of individuals with the pooled OR [43]. The funnel plot asymmetry and publication bias were assessed using Egger’s test and Begg’s test, respectively [44, 45]. The departure of frequencies of MTHFR rs1801131 A/C from expected values under HWE was assessed in controls by using the Pearson chi-square test. All statistical tests were performed using the Stata software (Version 11.0; StataCorp LP, College Station, TX).

The PolyPhen-2 bioinformatic tool was used to predict the effects of gene SNPs on the translated proteins. In the PolyPhen-2 analysis, the scores could range from 0 to 1, where a score of zero meant ‘benign’ and a score of one meant ‘probably damaging’.

### Network of gene-interaction of MTHFR gene

The network of gene-gene interaction for MTHFR gene was utilized through String online server (http://string-db.org/) [46].

## Results

### Study characteristics

**Fig. 1:**
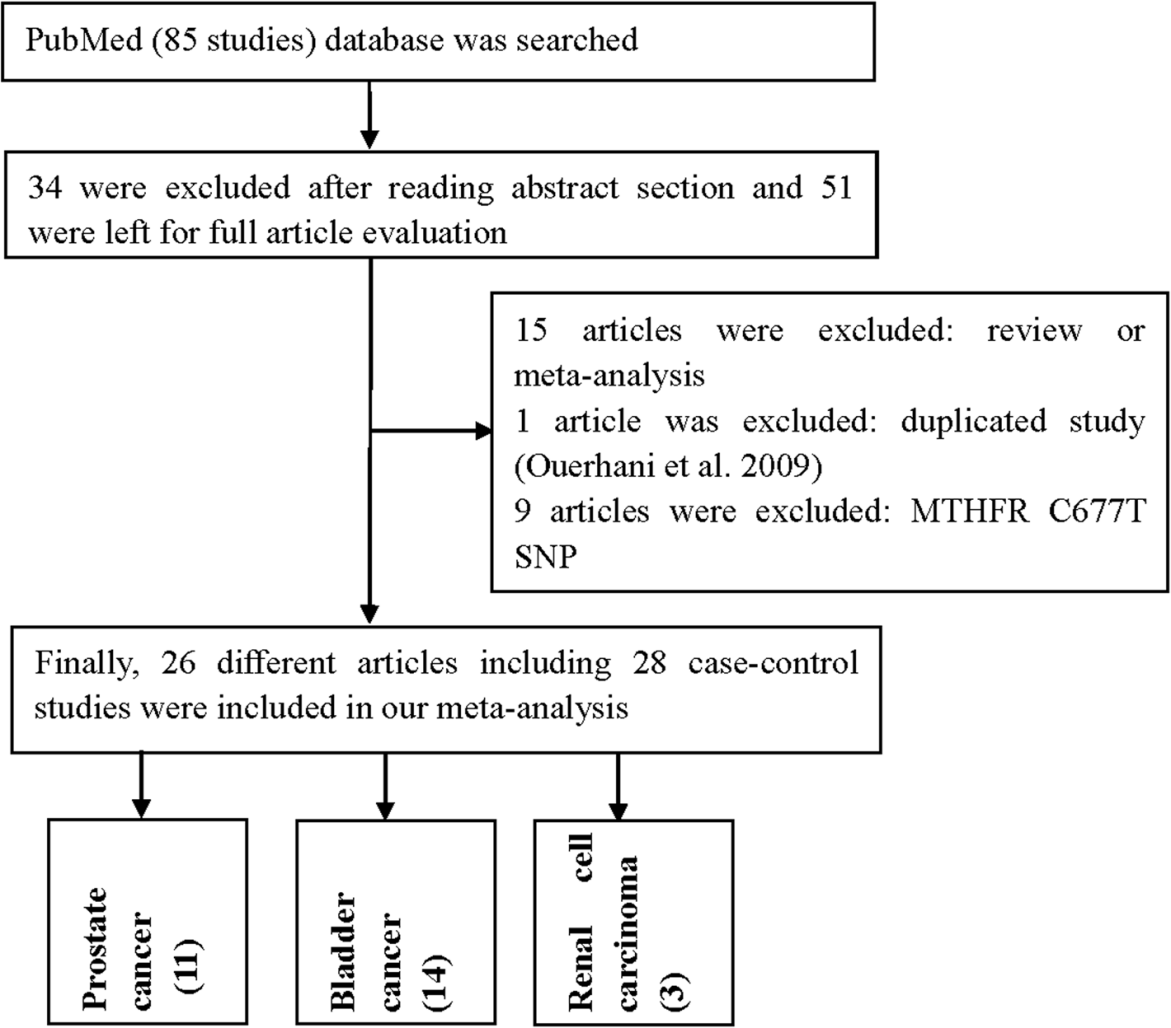
A flowchart illustrating the search strategy used to identify association studies for MTHFR rs1801131 polymorphism and urinary cancers’ risk

**Fig. 2:**
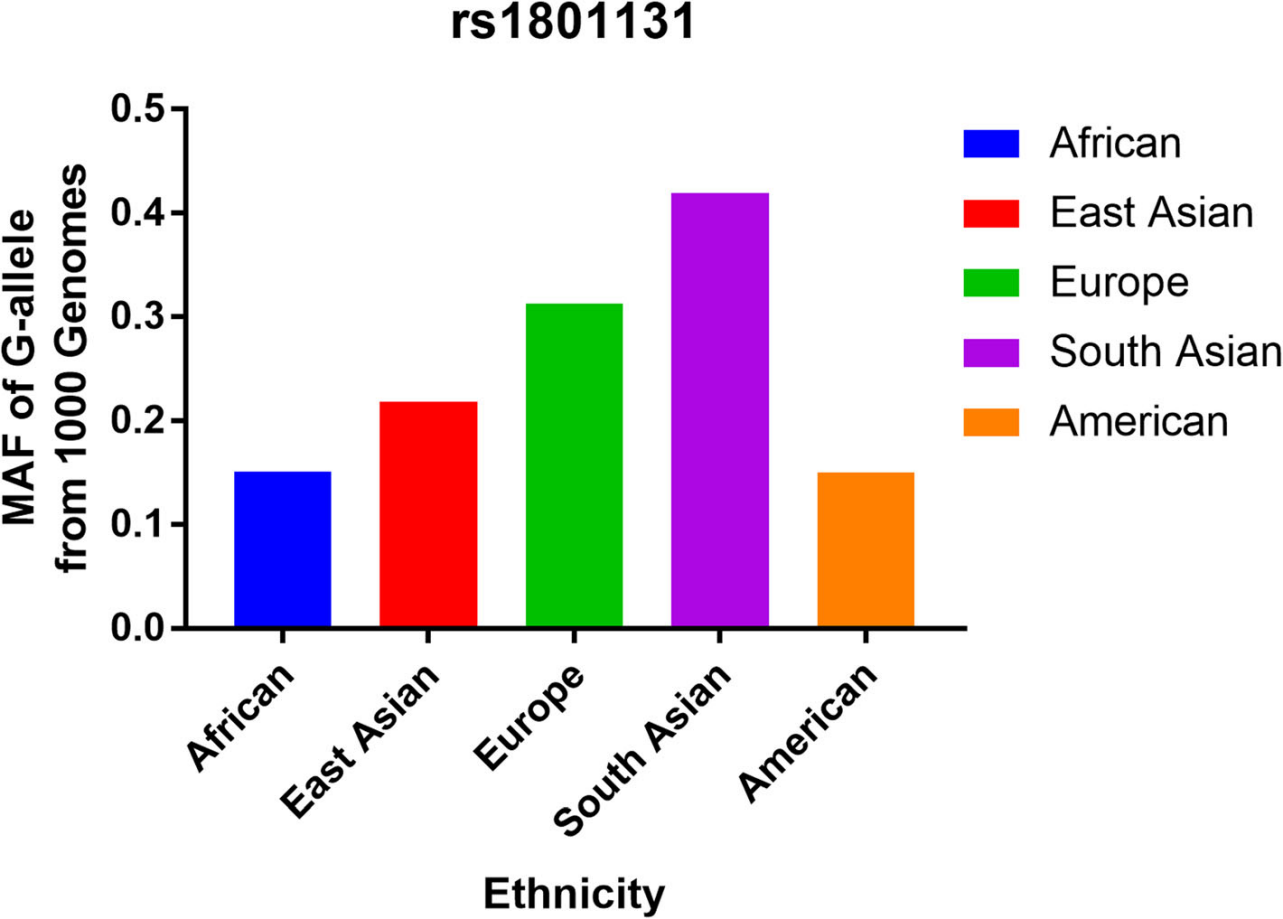
C-allele frequencies for the MTHFR gene rs1801131 polymorphism among cases/controls stratified by ethnicity. Vertical line, T-allele frequency; Horizontal line, ethnicity type. EAS: East Asian; EUR: European; AFR: African; AMR: American; SAS: South Asian

After reviewing the title, abstract, and full text, 51 different papers were included for the final analysis, expect for papers focusing on meta-analyses, reviews, case-only studies, and other gene polymorphisms. For bladder cancer, Ouerhani et al. published two papers in 2007 and 2009 that contained duplicated data about, so we included the larger numbers from Ouerhani (2007) et al. [24] in our analysis. Then, 15 different articles were review or meta-analysis. Moreover, another 9 papers were focus just only MTHFR C677T (rs1801133) polymorphism. Finally, we identified 26 different papers describing 28 case-control studies (11 case-control studies for prostate cancer, 14 for bladder cancer, and three for renal cell carcinoma, Table 1, Fig. 1) [9–34] to evaluate the association of MTHFR rs1801131 A/C. Study characteristics are shown in Table 1. The distribution of genotypes in the controls was consistent with HWE in all studies, except for three papers. The average NOS of including studies is 7.571, which means our results is credible and representational. None of the control populations had a history of malignant diseases. Genotyping methods were conducted using polymerase chain reaction and restrictive fragment length polymorphism (PCR-RFLP), and TaqMan technologies. Finally, we checked the Minor Allele Frequency (MAF) reported for the five main worldwide populations in the 1000 Genomes Browser (https://www.ncbi.nlm.nih.gov/snp/rs1801131#frequency_tab): East Asian (EAS), 0.219; European (EUR), 0.313; African (AFR), 0.151; American (AMR), 0.15; and South Asian (SAS), 0.42 (Fig. 2). The MAF in our analysis was 0.331 and 0.325 in the case and control group, respectively, both higher than the results in the EAS from1000 Genomes Browser database.

**Table 1:** Study characteristics of all included studies about urinary cancer

First author	Year	Origin	Ethnicity	Design	Source of control	Case	Control	Case			Control			HWE in control	Genotype method	NOS
								CC	CA	AA	CC	CA	AA			
Bladder cancer																
Ouerhani	2007	Tunisia	African	HB		111	131	6	47	58	9	37	85	0.55	PCR-RFLP	6
Rouissi	2009	Tunisia	African	HB		185	191	10	78	97	10	60	121	0.478	PCR-RFLP	7
Cai	2009	China	Asian	HB		312	325	6	91	215	7	92	226	0.504	PCR-RFLP	7
Izmirli	2011	Turkey	Caucasian	HB		47	50	3	25	19	7	29	14	0.195	PCR-RFLP	6
Safarinejad	2011	Iran	Caucasian	HB		158	316	25	85	48	23	115	178	0.46	PCR-RFLP	8
Lin	2004	USA	African	PB		21	21	0	7	14	0	8	13	0.281	PCR-RFLP	9
Wang	2009	China	Asian	PB		239	250	3	67	169	4	75	171	0.719	PCR-RFLP	9
Beebe-Dimmer	2012	USA	Caucasian	PB		218	272	14	109	95	34	111	127	0.211	Taqman	8
Karagas	2005	USA	Caucasian	PB		350	542	31	146	173	55	220	267	0.333	PCR-RFLP	9
Lin	2004	USA	Caucasian	PB		410	409	30	188	192	36	184	189	0.35	PCR-RFLP	9
Moore	2007	Spain	Caucasian	PB		1068	1078	74	457	537	92	429	557	0.467	TaqMan	7
Sanyal	2004	Germany	Caucasian	PB		311	245	33	133	145	24	111	110	0.6	PCR-RFLP	7
Lin	2004	USA	Mixed	PB		17	17	0	4	13	1	5	11	0.678	PCR-RFLP	9
Moore	2004	USA	Mixed	PB		106	108	9	45	52	8	45	55	0.771	TaqMan	8
Prostate cancer																
Cicek	2004	USA	Mixed	PB	Healthy	439	478	39	205	195	44	201	233	0.945	PCR-RFLP	8
Collin	2009	UK	Caucasian	PB	Healthy	1592	3035	144	673	775	289	1339	1407	0.249	PCR-RFLP	9
Cai	2010	China	Asian	HB	BPH	217	220	4	63	150	5	71	144	0.27	PCR-RFLP	6
Safarinejad	2010	Iran	Caucasian	HB	Healthy	174	348	14	70	90	40	150	158	0.628	PCR-RFLP	7
Singal	2004	USA	Caucasian	HB	BPH	81	42	9	43	29	7	17	18	0.396	PCR-RFLP	8
Wu	2010	Taiwan	Asian	HB	Healthy	218	436	10	70	138	14	135	287	0.697	PCR-RFLP	7
Marchal	2008	Spain	Caucasian	HB	Healthy	177	209	17	62	98	22	79	108	0.193	TaqMan	7
Stevens	2008	USA	Caucasian	PB	Healthy	1104	1109	105	518	481	125	493	491	0.94	TaqMan	7
Guelpen	2006	Sweden	Caucasian	PB	Healthy	222	434	27	108	87	55	203	176	0.765	TaqMan	7
Muslumanoglu	2009	Turkey	Caucasian	HB	BPH	91	166	44	16	31	44	45	77	< 0.05	PCR-RFLP	6
López-Cortés	2013	USA	Caucasian	PB	Healthy	104	110	2	2	100	1	1	108	< 0.05	PCR-RFLP	9
Renal cell carcinoma																
Ajaz	2012	Pakistan	Asian	HB		168	172	19	106	43	8	105	59	< 0.05	PCR-RFLP	6
Safarinejad	2012	Iran	Caucasian	PB		152	304	28	88	36	35	131	138	0.645	PCR-RFLP	9
Moore	2008	France	Caucasian	HB		818	1087	85	357	376	113	483	491	0.718	PCR-RFLP	7

*HB* hospital-based, *PB* population-based, *PCR-RFLP* polymerase chain reaction and restrictive fragment length polymorphism, *HWE* Hardy–Weinberg equilibrium, *NOS* Newcastle-Ottawa Score

### Quantitative synthesis

#### Total urinary cancers

**Fig. 3:**
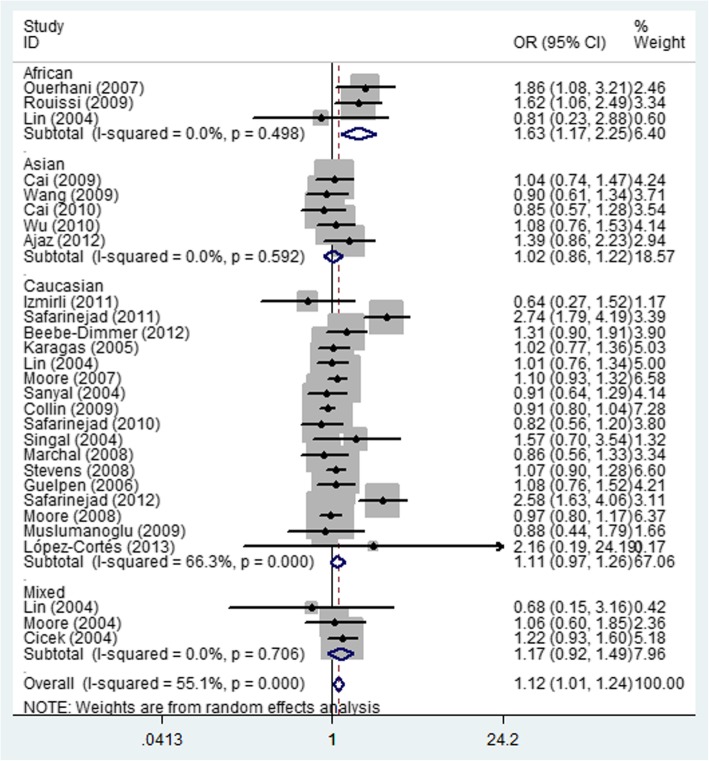
Forest plot of whole urinary cancers’ risk associated with the MTHFR rs1801131 polymorphism (CA vs. AA). The squares and horizontal lines correspond to the study-specific OR and 95% CI. The area of the squares reflects the weight (inverse of the variance). The diamond represents the summary OR and 95% CI

**Fig. 4:**
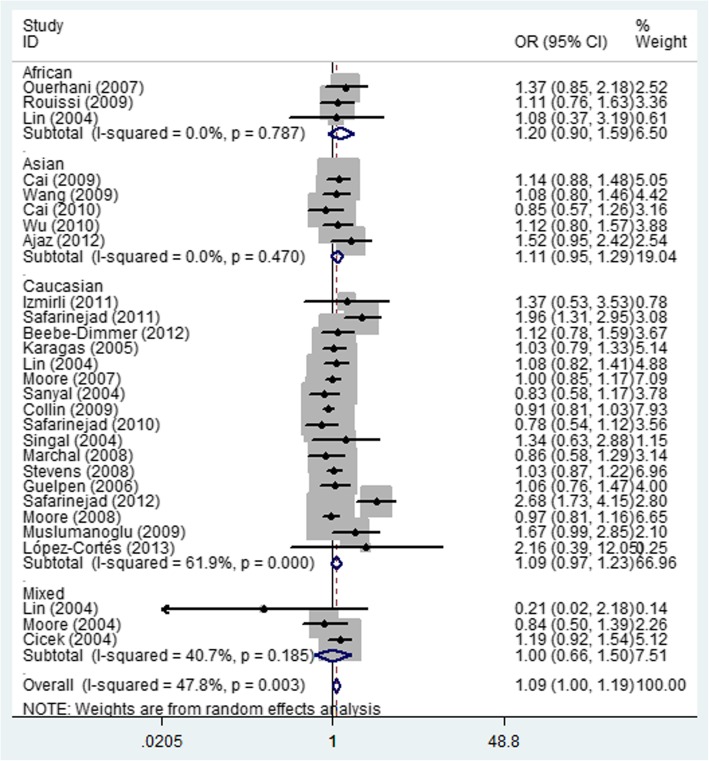
Forest plot of whole urinary cancers’ risk associated with the MTHFR rs1801131 polymorphism (CC + CA vs. AA). The squares and horizontal lines correspond to the study-specific OR and 95% CI. The area of the squares reflects the weight (inverse of the variance). The diamond represents the summary OR and 95% CI

In the total analysis, significant increased relationship was found in both heterozygote comparison (OR = 1.12; 95% CI = 1.01–1.24; *P* = 0.387 for heterogeneity, Fig. 3) and dominant genetic model (OR = 1.09; 95% CI = 1.00–1.19; *P* = 0.003 for heterogeneity, Fig. 4) between MTHFR rs1801131 A/C and urinary cancer risk. At the same time, if we excluded three papers that were not consistent with HWE, also similar association was detected (Table 2).

**Table 2:** Total and stratified analysis of MTHFR rs1801131 A/C polymorphism and each urinary cancer variables

Variables	N	Case/Control	C-allele vs. A-allele	CA vs. AA	CC vs. CA + AA	CC + CA vs. AA
			OR(95%CI) ***P***_**h**_	OR(95%CI) ***P***_**h**_	OR(95%CI) ***P***_**h**_	OR(95%CI) ***P***_**h**_
**Total**	28	9110/12105	1.06(0.98–1.15)0.000	**1.12(1.01–1.24)0.000**	1.01(0.87–1.17)0.021	**1.09(1.00–1.19)0.003**
**H**WE	25	8747/11657	1.03(0.96–1.11)0.001	**1.11(1.00–1.24)0.000**	0.93(0.84–1.02)0.432	1.06(0.98–1.16)0.006
**Prostate cancer**						
Total	11	4419/6587	1.02(0.91–1.14)0.016	0.99(0.91–1.07)0.569	1.00(0.81–1.25)0.062	0.99(0.91–1.07)0.253
HWE	9	4224/6311	0.96(0.91–1.02)0.656	0.99(0.91–1.07)0.423	0.90(0.79–1.04)0.918	0.97(0.90–1.05)0.461
Ethnicity						
Caucasian	8	3545/5453	1.02(0.88–1.17)0.008	0.96(0.88–1.06)0.622	0.96(0.83–1.10)0.020	0.96(0.88–1.05)0.244
Asian	2	435/656	1.02(0.81–1.27)0.250	0.97(0.75–1.27)0.392	1.23(0.61–2.48)0.546	0.99(0.77–1.29)0.300
Mixed	1	439/478	NA	NA	NA	NA
Source of control						
HB	6	958/1421	1.05(0.81–1.37)0.003	0.93(0.78–1.12)0.701	0.57(0.44–0.75)0.530	1.09(0.64–1.85)0.017
PB	5	3461/5166	0.97(0.91–1.04)0.485	1.00(0.91–1.10)0.269	1.08(0.85–1.36)0.004	0.91(0.79–1.06)0.885
BPH	3	389/428	1.22(0.70–2.13)0.004	0.95(0.68–1.31)0.411	1.22(0.44–3.40)0.310	1.12(0.84–1.50)0.116
Healthy	8	4030/6159	0.97(0.91–1.03)0.454	0.99(0.91–1.08)0.454	0.91(0.79–1.05)0.863	0.98(0.90–1.06)0.39
**Bladder cancer**						
Total	14	3553/3955	1.04(0.93–1.16)0.009	1.17(0.99–1.38)0.005	0.89(0.74–1.06)0.268	1.07(0.98–1.17)0.259
Ethnicity						
Caucasian	7	2512/2912	1.01(0.90–1.14)0.085	1.17(0.92–1.48)0.001	0.90(0.66–1.23)0.033	1.09(0.93–1.29)0.069
Asian	2	551/575	**1.35(1.15–1.60)0.941**	0.98(0.76–1.27)0.599	0.92(0.38–2.25)0.893	1.11(0.91–1.36)0.792
Mixed	2	123/125	0.75(0.51–1.09)0.286	1.00(0.59–1.70)0.594	1.04(0.41–2.65)0.469	0.78(0.48–1.27)0.258
African	3	317/343	0.91(0.72–1.14)0.627	**1.63(1.17–2.25)0.498**	0.88(0.44–1.75)0.702	1.20(0.90–1.59)0.787
Source of control						
HB	5	813/1013	1.16(0.90–1.49)0.024	1.49(0.96–2.30)0.002	1.24(0.84–1.84)0.137	**1.29(1.09–1.54)0.235**
PB	9	2740/2942	0.99(0.91–1.07)0.103	1.05(0.94–1.17)0.902	**0.82(0.67–0.99)0.788**	1.00(0.91–1.11)0.829
**Renal cell carcinoma**						
Total	3	1138/1563	1.33(0.90–1.98)0.000	1.47(0.81–2.68)0.000	1.50(0.86–2.59)0.041	1.54(0.82–2.92)0.000
HWE	2	970/1391	1.32(0.72–2.42)0.000	1.54(0.59–4.03)0.000	1.26(0.74–2.14)0.080	1.58(0.58–4.28)0.000

*Ph* value of Q-test for heterogeneity test, *NA* not available

#### Prostate cancer

Overall, there were no significant relationships between MTHFR rs1801131 A/C and prostate cancer risk in any of the available genotype models. Moreover, to avoid publishing bias, two papers that were not consistent with HWE were excluded, so 9 case-control studies were left for analysis, and, to our regret, no association was also detected. Finally, based on ethnicity-stratified and source of control subgroup analysis, there remain no significant association were found (Table 2).

#### Bladder cancer

**Fig. 5:**
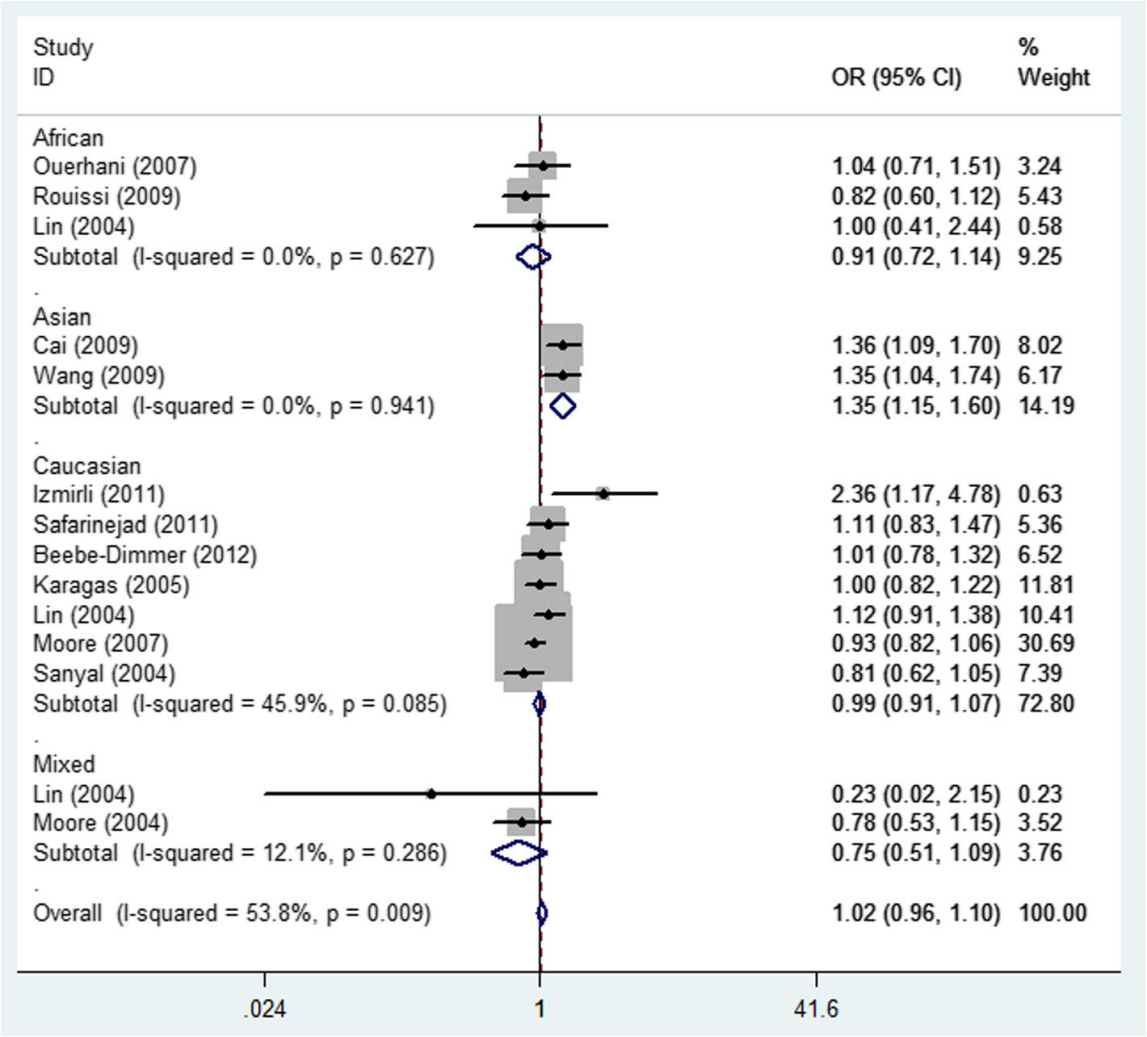
Forest plot of bladder cancer risk associated with the MTHFR rs1801131 polymorphism (C-allele vs. A-allele) by ethnicity subgroup. The squares and horizontal lines correspond to the study-specific OR and 95% CI. The area of the squares reflects the weight (inverse of the variance). The diamond represents the summary OR and 95% CI

**Fig. 6:**
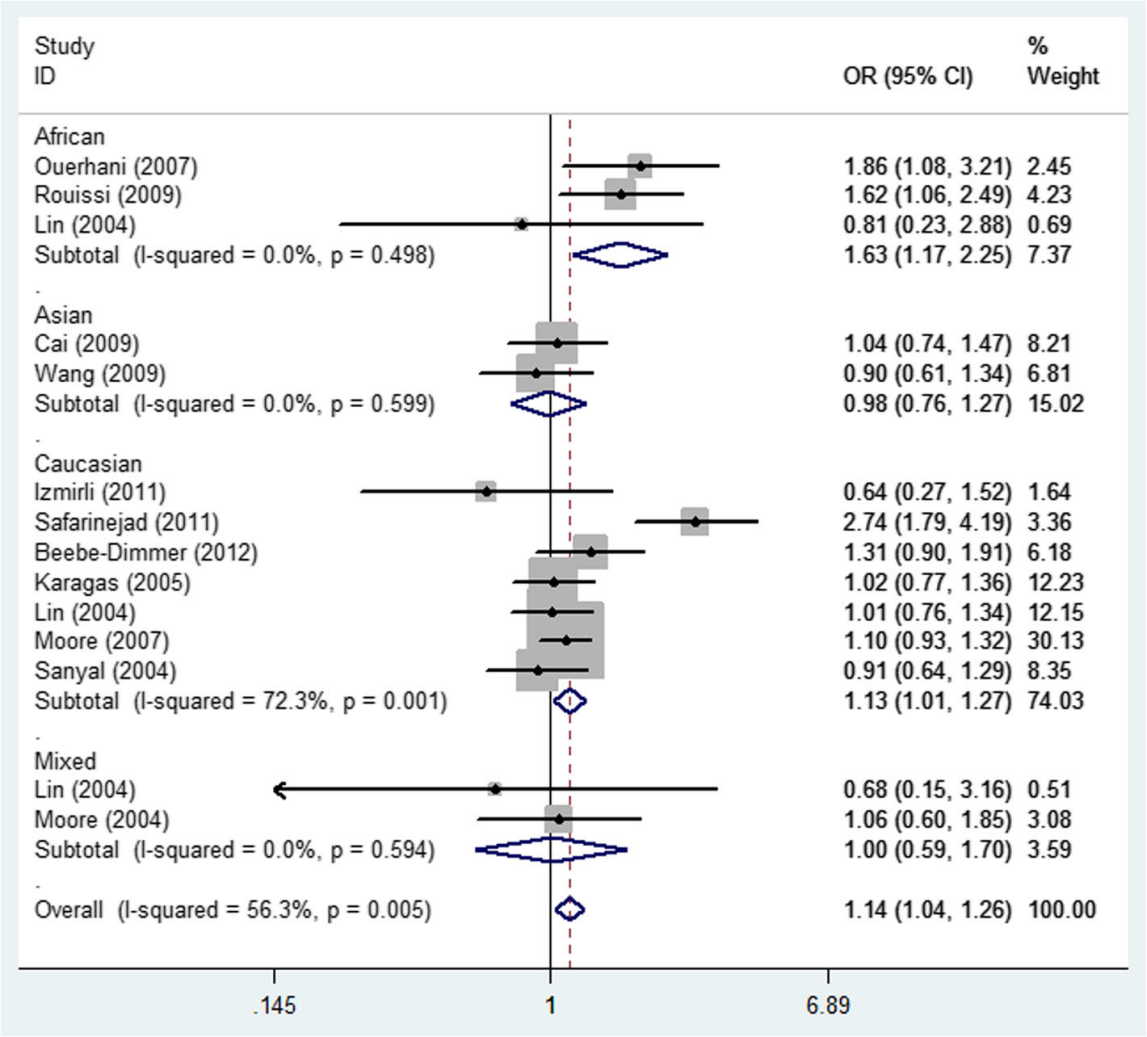
Forest plot of bladder cancer risk associated with the MTHFR rs1801131 polymorphism (CA vs. AA) by ethnicity subgroup. The squares and horizontal lines correspond to the study-specific OR and 95% CI. The area of the squares reflects the weight (inverse of the variance). The diamond represents the summary OR and 95% CI

**Fig. 7:**
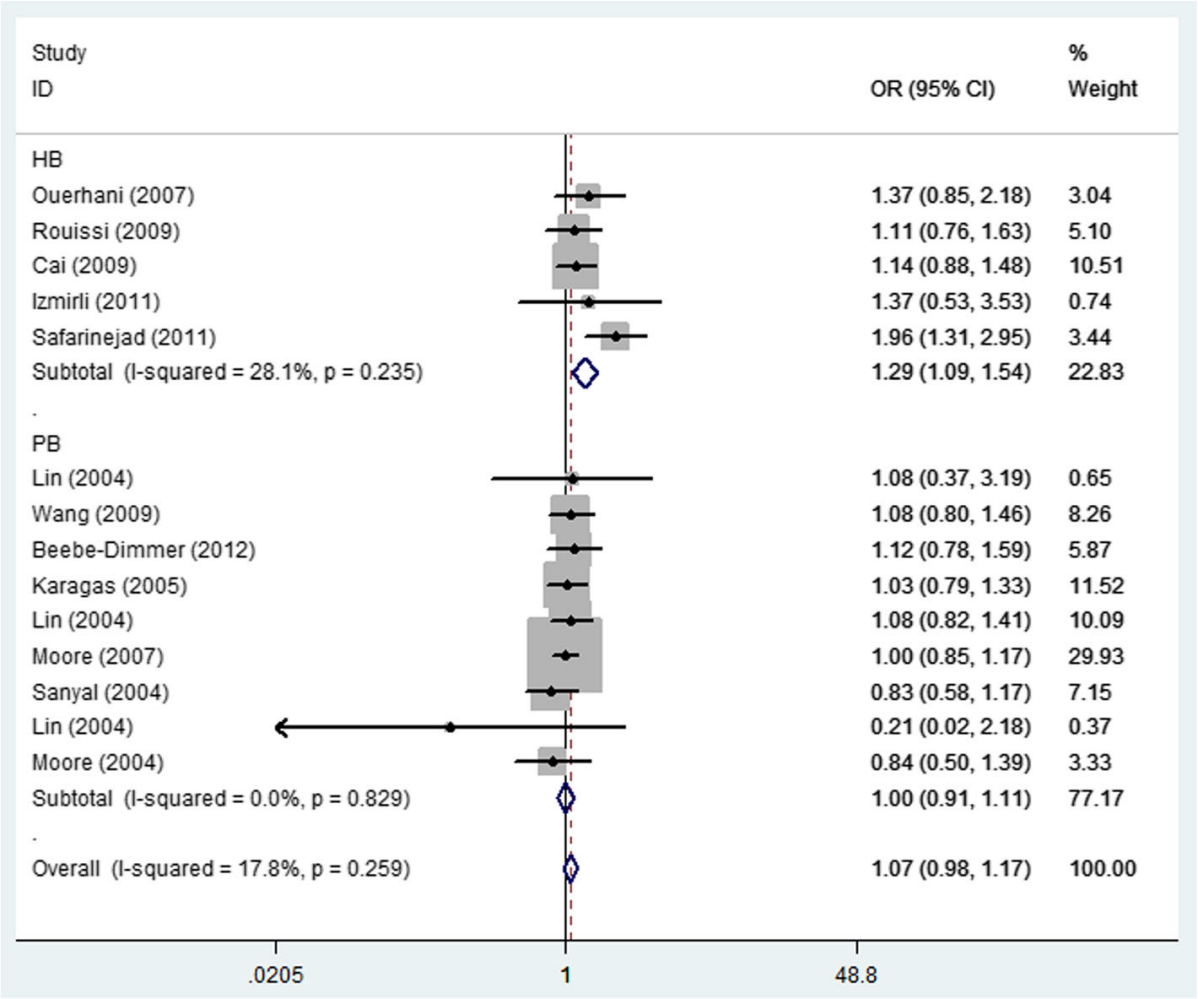
Forest plot of bladder cancer risk associated with the MTHFR rs1801131 polymorphism (CC + CA vs. AA) by source of control subgroup. The squares and horizontal lines correspond to the study-specific OR and 95% CI. The area of the squares reflects the weight (inverse of the variance). The diamond represents the summary OR and 95% CI

Detailed results of the meta-analysis are shown in Table 2. No statistically significant association was detected between MTHFR rs1801131 A/C and bladder cancer risk in the total group or in the all articles according to HWE. Interestingly, in the ethnicity subgroup analysis, there was a increased risk of bladder cancer in the Asian population (allelic contrast: OR = 1.35, 95% CI = 1.15–1.60, *P*_heterogeneity_ = 0.941, Fig. 5), and African population (heterozygote comparison: OR = 1.63, 95% CI = 1.17–2.25, *P*_heterogeneity_ = 0.498, Fig. 6), but not in Caucasians, or Mixed (Table 2). Moreover, in the subgroup analysis in source of control, also increased relationship was detected in dominant genetic model (OR = 1.29, 95% CI = 1.09–1.54, *P*_heterogeneity_ = 0.235, Fig. 7).

#### Renal cell carcinoma

In the total and only HWE analysis, no increased relationship was found between MTHFR rs1801131 A/C and renal cell carcinoma (Table 2).

### Meta-regression

**Fig. 8:**
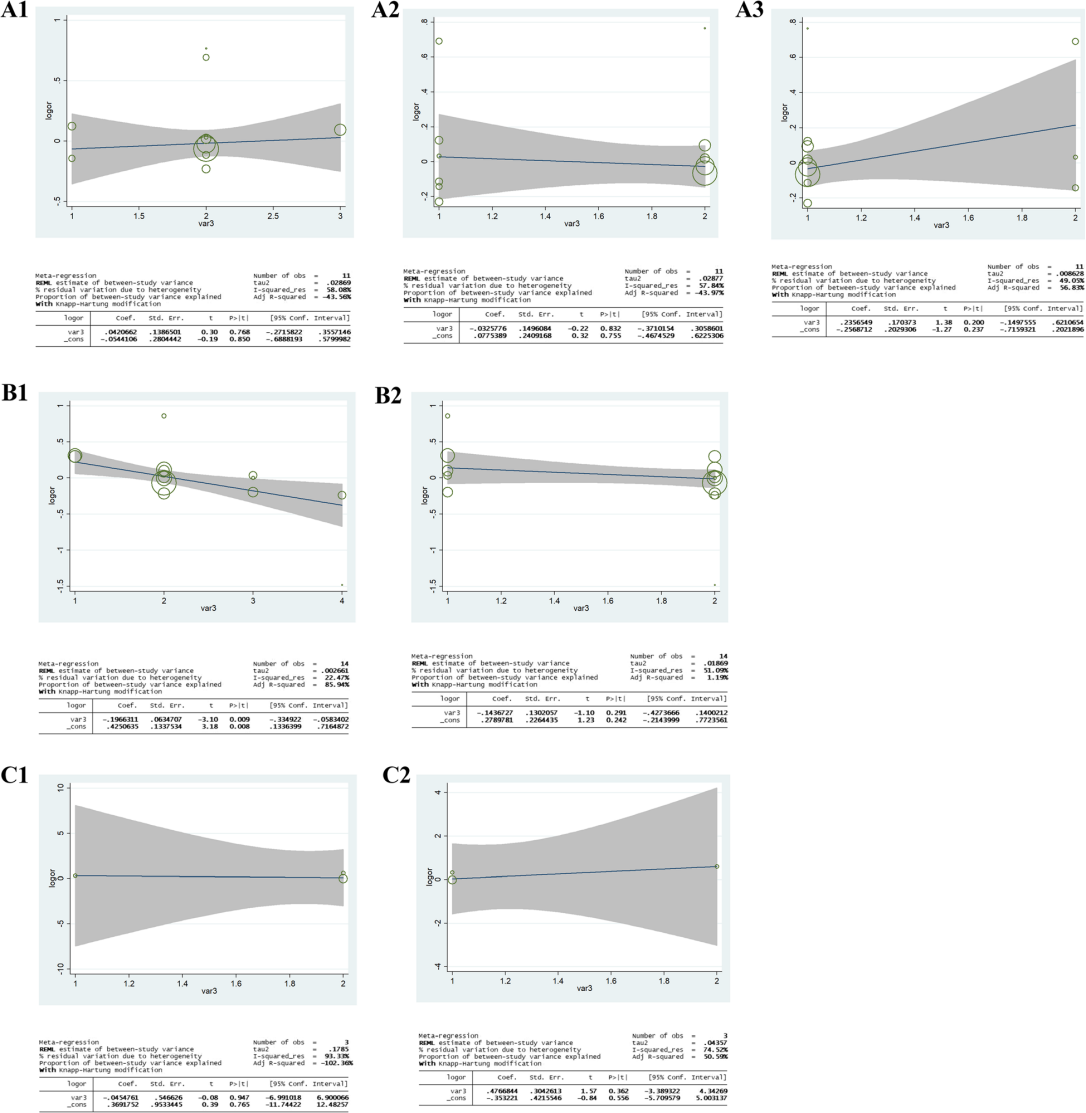
Random-effect meta-regression of log odds ratio versus ethnicity (A1), source of control (A2), control type (A3), respectively in prostate cancer. Random-effect meta-regression of log odds ratio versus ethnicity (B1), source of control (B2), respectively in bladder cancer. Random-effect meta-regression of log odds ratio versus ethnicity (C1), source of control (C2), respectively in renal cell carcinoma

Considering the subgroup of ethnicity, source of control, and control type as independent variables and the log (OR) as dependent variable, the random-effect meta-regression results were presented in Fig. 8. To estimate the functional relationship of the log OR with above three items, the analysis showed only a significant relationship for allele model (C-allele vs. A-allele) for the ethnicity with a regression coefficient of 0.009 in bladder cancer, rather than other subgroups and other urinary cancers, which means the heterogeneity for rs1801131 polymorphism in bladder cancer may be from the subgroup of ethnicity.

### Publication bias diagnosis and sensitivity analysis

**Fig. 9:**
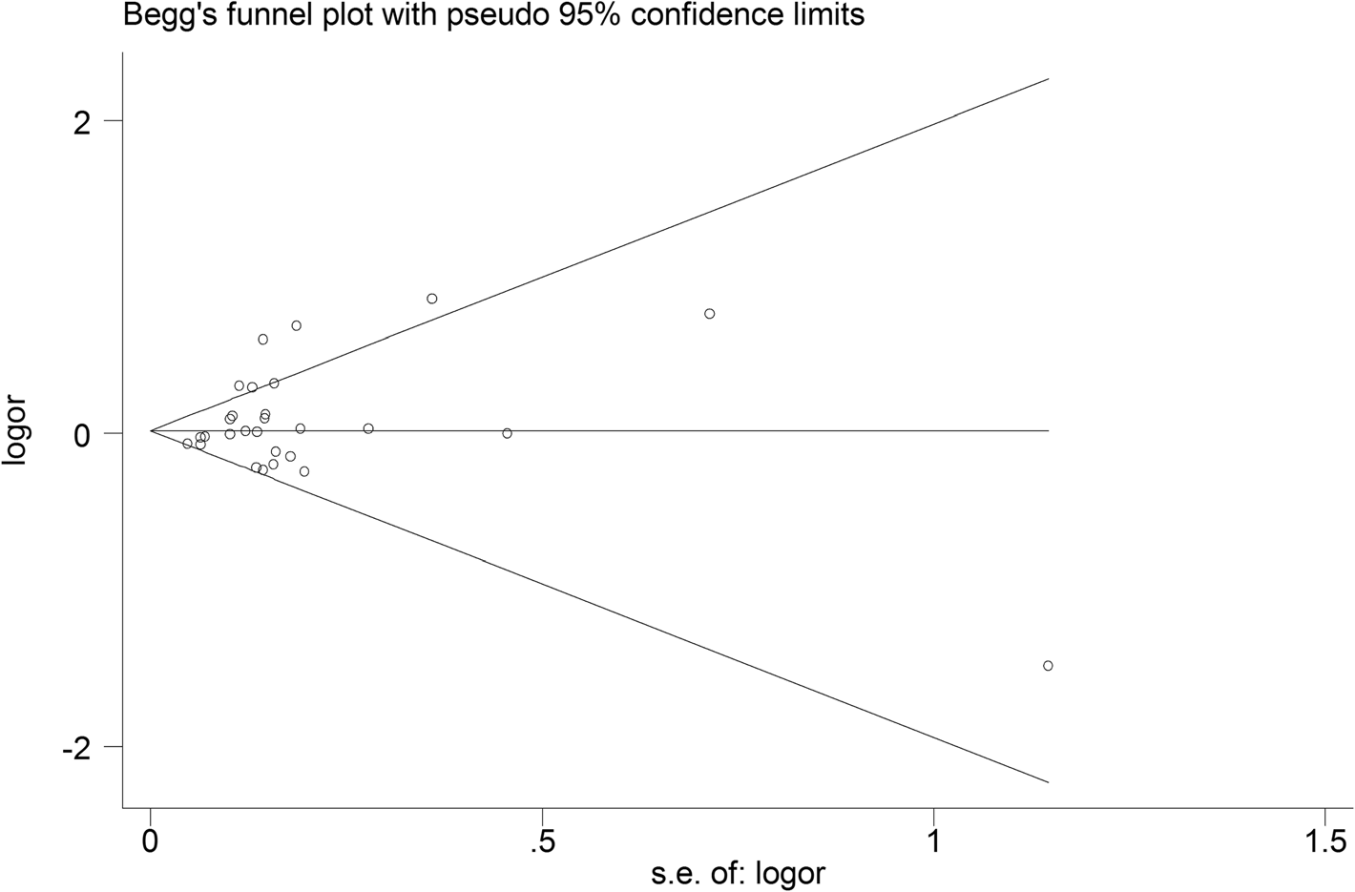
Begg’s funnel plot for publication bias test (CA vs. AA). Each point represents a separate study for the indicated association. Log [OR], natural logarithm of OR. Horizontal line, mean effect size

**Fig. 10:**
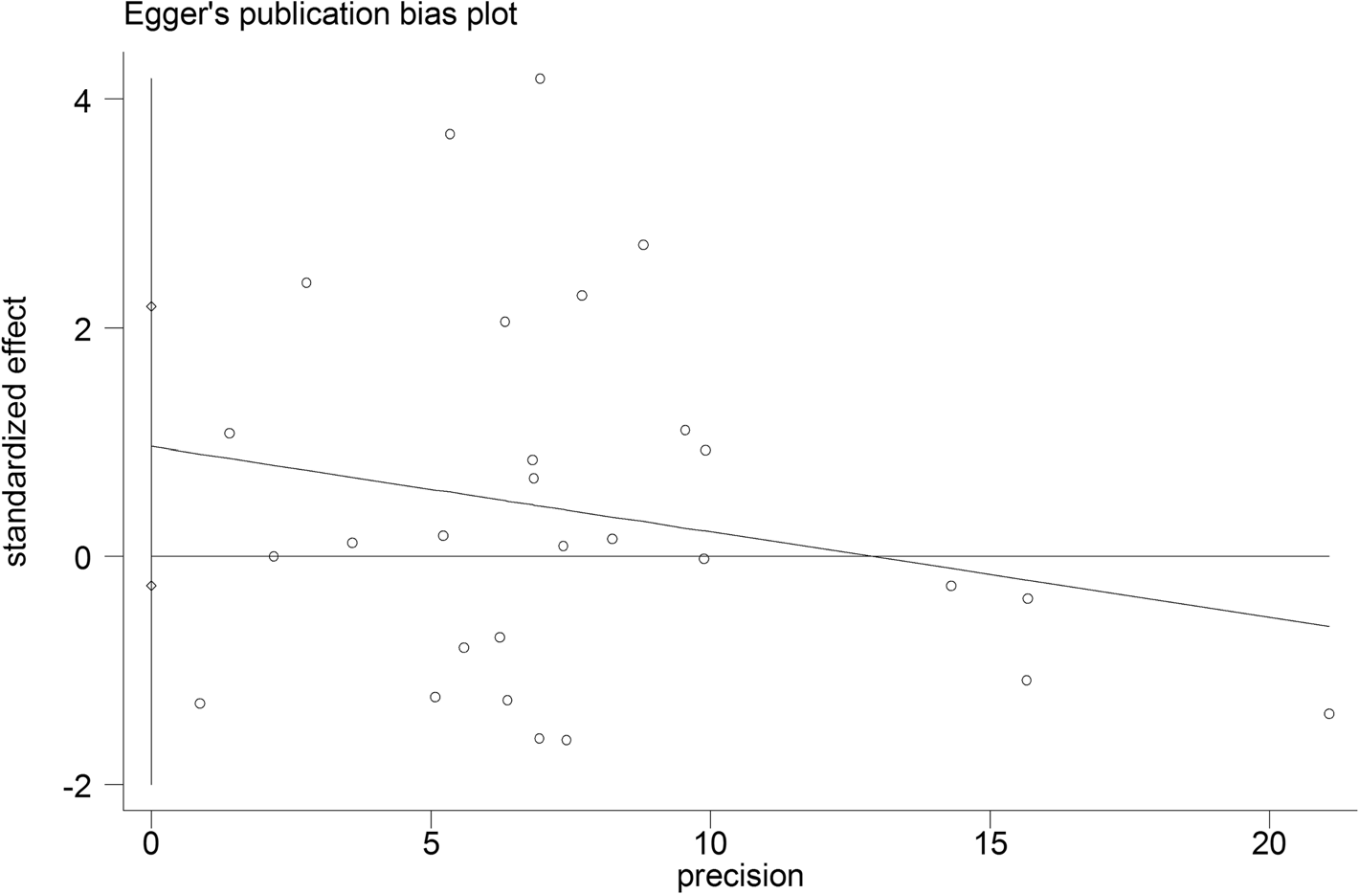
Egger’s publication bias plot (CA vs. AA). Each point represents a separate study for the indicated association. Horizontal line, mean effect size

**Fig. 11:**
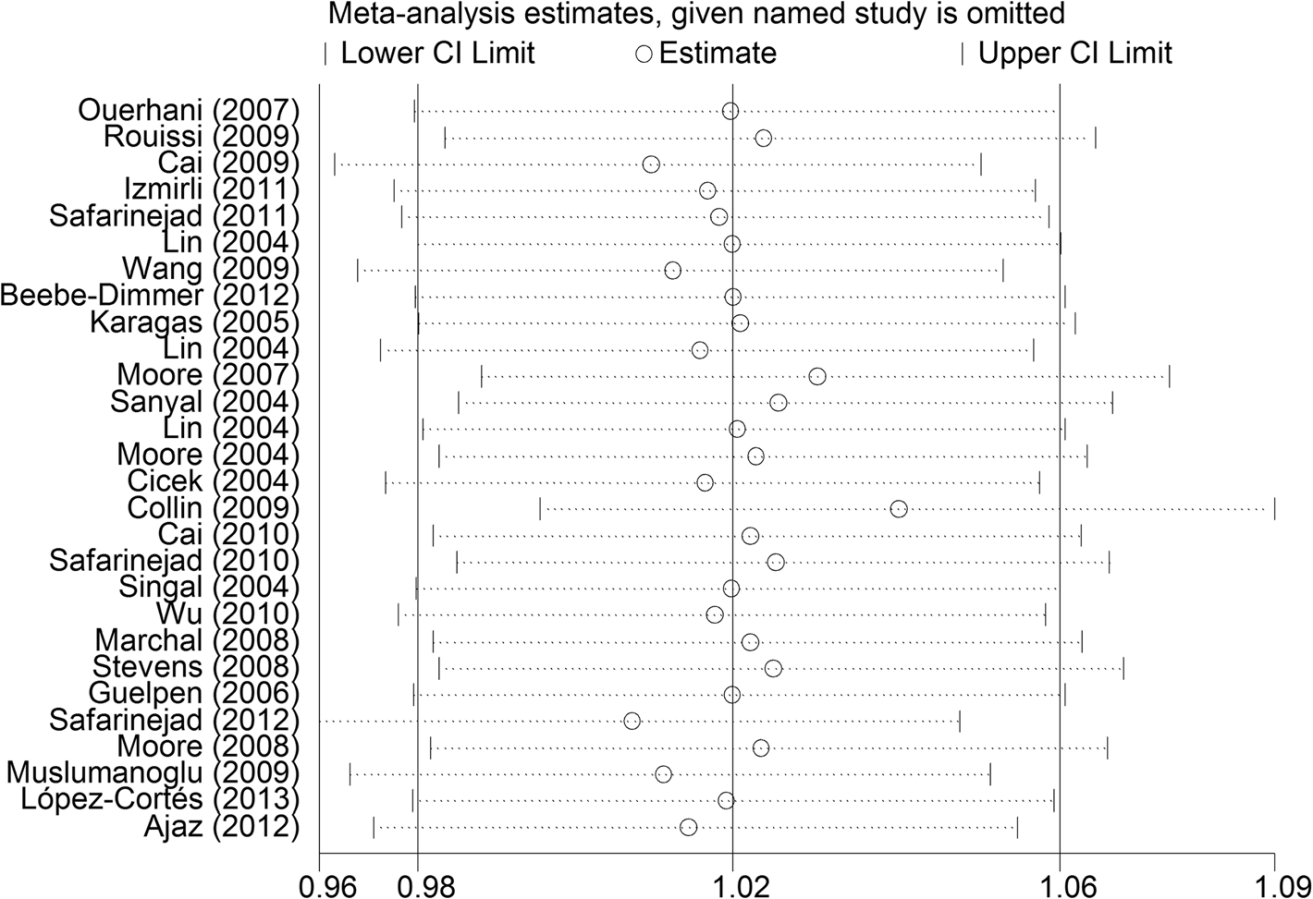
Sensitivity analysis between the MTHFR rs1801131 polymorphism and whole urinary cancers’ risk (C-allele vs. A-allele)

Begg’s funnel plot and Egger’s test were performed to access the publication bias of the literature. The shape of the funnel plot did not reveal obvious asymmetry and the Egger’s test suggested the absence of publication bias [for example (CA vs. AA) (z = 1.61, *P* = 0.119 for Begg’s test; t = 1.01, *P* = 0.314 for Egger’s test, Figs. 9, 10)]. Instead of above, we also deleted each study involved in our meta-analysis to reflect the influence of the individual data-set on the pooled OR, then the corresponding pooled OR was not significantly altered, indicating that our results were statistically robust (for example: allelic contrast, Fig. 11).

### PolyPhen-2 analysis

**Fig. 12:**
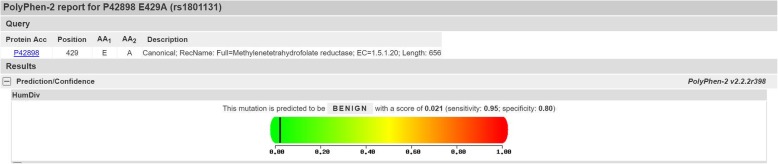
Analysis of the effect of rs1801131 polymorphism on the MTHFR protein using the Polyphen-2 bioinformatics tool. The position of the black line represents the score, and a measure of how damaging the mutation could be as the protein function

To verify this association, we used the PolyPhen-2 tool to analyze the features of the rs1801131 mutant. A score of 0.021 was obtained from the analysis, suggesting the possibility of rs1801131 not being a damaging mutation (Fig. 12).

### Gene-gene interaction of online analysis

**Fig. 13:**
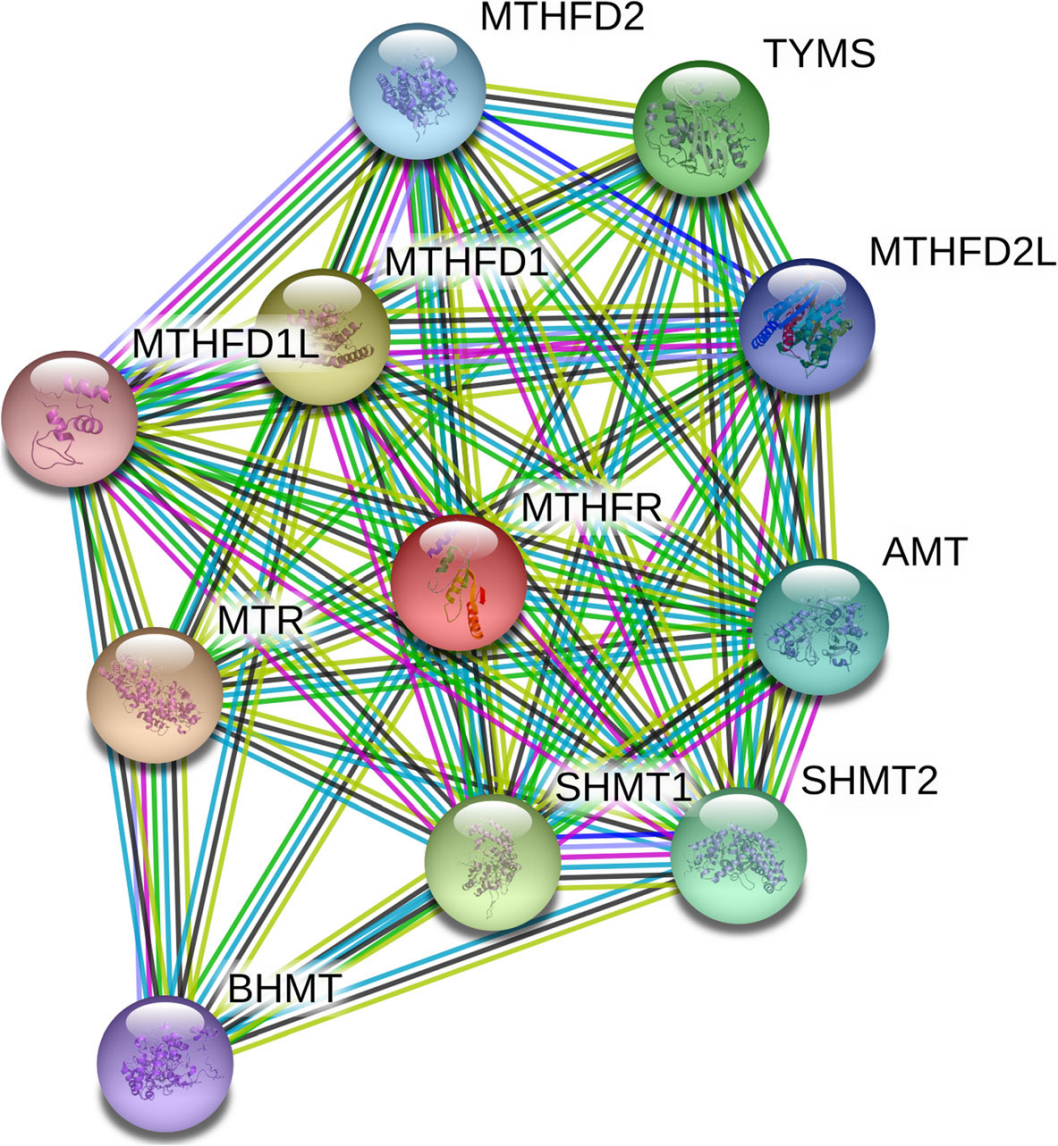
Human MTHFR interactions network with other genes obtained from String server. At least 10 genes have been indicated to correlate with MTHFR gene. MTR: 5-methylterahydrofolate-homocysteine methyltransferase; MTHFD: Methylenetetrahydrofolate dehydrogenase (NADP+ dependent); SHMT1: Serine hydroxymethyltransferase 1(soluble); TYMS: Thymidylate synthetase; SHMT2: Serine hydroxymethyltransferase 2 (mitochondrial); AMT: Aminomethyltransferase; MTHFD2L: MTHFD 2-like (347 aa); BHMT: Betaine-homocysteine S-methyltransferase; MTHFD1L: MTHFD 1-like

String online server indicated that MTHFR gene interacts with numerous genes. The network of gene-gene interaction has been illustrated in Fig. 13.

## Discussion

Our study was focused on the MTHFR rs1801131 polymorphism. The mutant C-allele of the MTHFR rs1801131 polymorphism has been reported to reduce the MTHFR enzymatic activity of the wild type A-allele [8], which may increase cancer risk. For example, Safarinejad et al. [27] reported that reduced levels of MTHFR mRNA had an increased association with the risk in men bladder cancer, which may be explained by the hypothesis that reduced MTHFR mRNA level may influence the metabolism of folic acid, then decrease supply of 5-MTHF in serum, along with the increase other forms of folic acid, which leads to affect the synthesis of the pyrimidine and purine, resulting in damaged in DNA synthesis and repair, finally contributes to cancer development.

This is the first meta-analysis to estimate the relationship between MTHFR rs1801131 and urinary cancers’ risk, involving approximately 9110 cancer cases and 12,105 controls. Increased associations were found between this polymorphism and urinary cancers. Moreover, in the specific bladder cancer, this polymorphism was associated with increased bladder cancer’s susceptibility in Asians and Africans, but not Caucasians, in some different genetic models. The classic five genetic models were applied very popular and credible. If one of five model is significant, this group is considered as positive association. Additional, between different subgroups, such as ethnicity, it is normal that the associations were detected in different genetic models or the same models, because different items were existed among the groups. The polymorphism may act as a risk factor in urinary cancers, especially bladder cancer, possibly through the mechanism described above.

Interestingly, previous two meta-analysis reported that another MTHFR rs1801133 (C677T) had a decreased association in whole cancer risk and urinary cancers [47, 48]. Above two different polymorphisms in the same MTHFR gene had the complete opposite function. Following reasons may explain above results. First, different polymorphism sites may have the opposite effect on the expression of its host gene. Second, cancer is a complex disease, and may not be depended entirely on a gene or one kind of polymorphism, moreover, gene-gene or gene-environment factors may play a significant influence on the susceptibility of urinary cancers [49].

In addition, we used the online analysis system-String to predict potential and functional partners (Fig. 12). Finally, 10 genes were predicted. The highest score of association was MTR (Score = 0.999), however, MTHFD1L was the last in line (Score = 0.896). Enzymes in one-carbon metabolism genes, such as MTR, MTHFD, TYMS, SHMT, MTHFR can both regulate the metabolism of folate, and low folate levels can induce carcinogenesis [50–53]. First, polymorphisms in MTR gene increase homocysteine in the plasma, resulting in changes to the folate pathway and increasing association of carcinogenesis [54, 55]. Second, MTHFD polymorphisms (G1958A and T401C) had a strong association with total plasma homocysteine levels and gastric cancer risk and were modulated by genotypes of MTHFR simultaneously [56]. Third, the rs3819102 polymorphism in TYMS might increase susceptibility to the risk of lung cancer [57]. Fourth, the SHMT1 C1420T polymorphism was associated with decreased risk of breast cancer [58]. Above information predicted one-carbon metabolism genes: MTHFR and others may influence different kinds of tumors’ development, which maybe become intervention and treatment target genes in the future.

There are some limitations inherent in the included studies. First, despite inclusion of all the eligible studies, the resultant sample size is still not large enough; this situation may increase the likelihood of type I and type II errors. Second, we just searched articles from Pubmed, some other studies maybe omitted. Third, the cancer may not be depended entirely on a gene or one kind of polymorphism, because different results were found in rs1801131 polymorphism, and in different SNPs (such as rs1801133 polymorphism) in the same MTHFR gene in current analysis, further studies should be to identified more valuable and credible polymorphisms. Fourth, it is necessary to evaluate the roles of some special environmental factors (such as age, gender, the body-mass index, diet, alcohol consumption, smoking status) and lifestyles. Fifth, significant associations were detected in different genetic models in the same subgroup, this inconsistency may indicate the influence of type I error by the repetitive comparison.

In summary, our present update analysis suggested novel evidence that the MTHFR rs1801131 polymorphism has a risk effect for urinary cancers, especially bladder cancer. Further studies with larger samples, are needed to evaluate associations between MTHFR rs1801131 polymorphism and urinary cancers’ risk.

## Data Availability

All the data generated in the present research is contained in this manuscript.

## Abbreviations

MTHFR: Methylenetetrahydrofolate reductase
5,10-MTHF: 5,10-methylenetetrahydrofolate
SAM: S-adenosylmethionine
ORs: Odds ratios
CIs: Confidence intervals
SNPs: Single nucleotide polymorphisms
HWE: Hardy-Weinberg equilibrium
